# Ikaros Regulates microRNA Networks in Acute Lymphoblastic Leukemia

**DOI:** 10.3390/epigenomes6040037

**Published:** 2022-10-19

**Authors:** Sophie Kogut, Hana Paculova, Princess Rodriguez, Joseph Boyd, Alyssa Richman, Amrita Palaria, Hilde Schjerven, Seth Frietze

**Affiliations:** 1 Department of Biomedical and Health Sciences, University of Vermont, Burlington, VT 05405, USA; 2 Department of Microbiology and Molecular Genetics, University of Vermont, Burlington, VT 05405, USA; 3 Cellular Molecular Biomedical Sciences Program, University of Vermont, Burlington, VT 05405, USA; 4 Department of Laboratory Medicine, University of California, San Francisco, CA 94143, USA; 5 Helen Diller Family Comprehensive Cancer Center, University of California San Francisco, San Francisco, CA 94143, USA; 6 The University of Vermont Cancer Center, Burlington, VT 05405, USA

**Keywords:** miRNA, IKZF1, acute lymphoblastic leukemia

## Abstract

The hematopoietic transcription factor Ikaros (*IKZF1*) regulates normal B cell development and functions as a tumor suppressor in precursor B cell acute lymphoblastic leukemia (B-ALL). MicroRNAs (miRNAs) are small regulatory RNAs that through post-transcriptional gene regulation play critical roles in intracellular processes including cell growth in cancer. However, the role of Ikaros in the regulation of miRNA expression in developing B cells is unknown. In this study, we examined the Ikaros-regulated miRNA targets using human *IKZF1*-mutated Ph^+^ B-ALL cell lines. Inducible expression of wild-type Ikaros (the Ik1 isoform) caused B-ALL growth arrest and exit from the cell cycle. Global miRNA expression analysis revealed a total of 31 miRNAs regulated by IK1, and ChIP-seq analysis showed that Ikaros bound to several Ik1-responsive miRNA genes. Examination of the prognostic significance of miRNA expression in B-ALL indicate that the IK1-regulated miRNAs hsa-miR-26b, hsa-miR-130b and hsa-miR-4649 are significantly associated with outcome in B-ALL. Our findings establish a potential regulatory circuit between the tumor-suppressor Ikaros and the oncogenic miRNA networks in *IKZF1*-mutated B-ALL. These results indicate that Ikaros regulates the expression of a subset of miRNAs, of which several may contribute to B-ALL growth.

## 1. Introduction

Acute lymphoblastic leukemia (ALL) is a leading cause of childhood cancer-related mortality and is also a significant health challenge to adults [1]. The majority of childhood ALL cases are of the precursor B-cell subtype of acute leukemia (B-ALL) and can be characterized by recurrent genomic lesions. The Philadelphia chromosome (Ph^+^) results from the t(9;22)(q34;q11) translocation involving the *BCR* and *ABL1* genes (referred to as Ph^+^ B-ALL) which leads to constitutive activation of the ABL1 kinase [2,3]. Significant improvements in the diagnosis and treatment for Ph^+^ B-ALL have been made in the past few decades and have resulted in a survival rate of >90% [4]. For example, the combination of chemotherapy with tyrosine kinase inhibitors (TKIs) have proven effective for the treatment of Ph^+^ B-ALL [3,5]. However, despite the improved outcomes observed in childhood B-ALL cases, up to 20% of patients still relapse [4,6]. In addition, patients older than 40 years old have not greatly benefited from current treatments and their 5-year overall survival remains lower than 45% [7,8]. Improved understanding of the disease mechanisms associated with poor outcome B-ALL subtypes will enable the identification of new biomarkers and molecular targets for drug development.

Genome sequencing analysis has identified cooperating gene mutations in B-ALL, many encoding for hematopoietic transcription factors (TFs) known to be critical regulators of B-cell development [9,10,11,12]. In Ph^+^ B-ALL patients, this includes mutations in *IKZF1* (encoding Ikaros) (80%), *PAX5* (51%), and *EBF1* (13%) [13,14]. Of the genes mentioned, a role for the *IKZF1* gene as a tumor suppressor has been established in both pediatric and adult leukemia, where a number of high-risk B-ALL subtypes harbor *IKZF1* deletions or mutations [12,15,16]. Greater than 80% of Ph^+^ B-ALL cases exhibit mutations or deletions in the *IKZF1* gene [14]. In addition, *IKZF1* mutations are common in the high-risk B-ALL subtype termed “Ph-like”, which has a gene expression profile similar to Ph^+^ B-ALL [17]. In comparison to the full-length Ikaros molecule (termed the Ik1 isoform), intragenic deletions of the *IKZF1* exons 4 to 7 encoding the DNA-binding zinc fingers are common in these cases and result in the expression of a dominant negative Ikaros isoform (termed Ik6) [14,18,19]. Inactivating mutations of *IKZF1* are strongly associated with a poor prognosis in different subtypes of childhood B-ALL [12]. Despite an established tumor suppressor role for Ikaros, there is still a significant gap in our understanding of the molecular mechanisms of precursor B cell oncogenesis and the molecular targets of Ikaros in B-ALL. 

MicroRNAs (miRNAs) are small endogenous RNAs that regulate development and hematopoiesis via post-transcriptional gene silencing [20]. miRNAs inhibit the translation or induce mRNA degradation of select target mRNAs to modulate diverse cellular processes, including cell growth, apoptosis, differentiation and proliferation [21,22]. Humans are known to express thousands of different miRNAs. Accumulating evidence indicates that miRNAs are implicated in the pathogenesis and progression of cancer. For example, different miRNAs can activate or suppress oncogenic phenotypes by inhibiting the expression of tumor suppressor genes or oncogenes, respectively [23,24]. Commonly, oncogenic miRNAs (oncomiRs) are overexpressed in cancers while tumor-suppressive miRNAs (tsmiRs) are lowly expressed. miRNAs have additionally been identified as candidates for diagnostic and prognostic biomarkers, as well as predictors of drug responses in cancer [25,26]. Importantly, miRNAs represent candidate clinical biomarkers that may act as indicators of relapse for B-ALL [26,27,28]. However, there have been limited number of such studies and new clinically relevant biomarkers are still needed. 

Here, we investigated a role of Ikaros in the regulation of miRNA expression in B-ALL cells. We used *IKZF1*-mutated B-ALL cells with inducible expression of wild-type Ikaros to identify miRNAs that change expression with Ikaros-induced growth arrest. We determined the binding of Ikaros to the genomic regions of differentially regulated miRNA genes, as well as the prognostic significance of Ikaros-regulated miRNA expression in a patient cohort of B-ALL. Our results indicate that Ikaros regulates the expression of a subset of miRNAs, providing new insight into potential B-ALL growth mechanisms.

## 2. Results

### 2.1. Inducible Expression of IK1 Results in Growth Arrest in IKZF1-Mutated B-ALL Cells 

**Figure 1:**
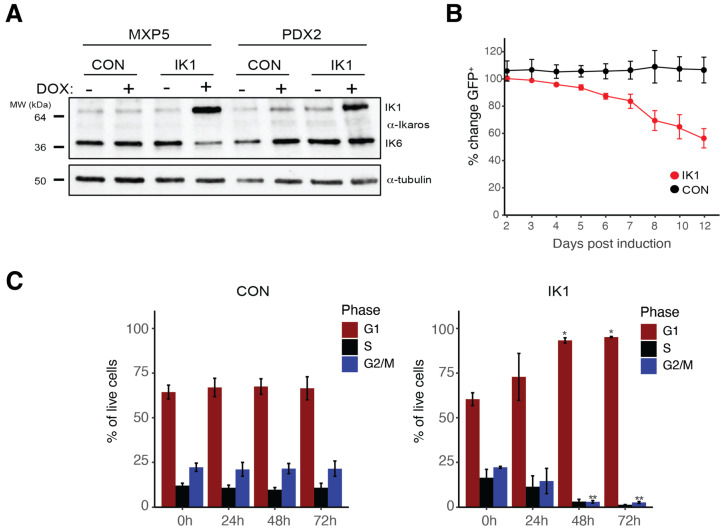
Ikaros induction in B-ALL cells reduces cell cycle. (**A**) Immunoblot analysis of Ikaros in *IKZF1*-deleted Ph^+^ B-ALL cells (MXP5 and PDX2) with stable expression of doxycycline (DOX) inducible empty vector (CON) or IK1 following 24 h of induction. IK1 and IK6 isoforms are indicated, α-tubulin was used as a loading control. (**B**) Growth competition analysis comparing the percentage of viable GFP^+^ MXP5 cells following DOX induction of CON and IK1 over the period of 12 days, starting with >95% GFP^+^ cells. Error bars, SEM; *n* = 3, significant differences were observed between IK1 and CON for all timepoints except day 2, *p* < 0.05; *t*-test. (**C**) Effect of IK1 induction on cell cycle distribution analyzed using flow cytometry, representing the percentage of cell populations in CON (left) and IK1 (right) cells. Error bars indicate standard deviation, *n* = 2, (*, *p* < 0.05; **, *p* < 0.005; *t*-test versus 0 h timepoint).

Mutations in *IKZF1* are charateristic of Ph^+^ ALL and are associated with poor prognosis [12,14]. MXP5 and PDX2 are human Ph^+^ B-ALL cell lines that harbor a heterozygous intragenic deletion in the *IKZF1* gene resulting in the expression of the dominant negative Ik6 isoform of Ikaros [14,29]. Our prior studies in PDX2 cells had showed that ectopic expression of wild-type Ikaros (IK1) inhibited cell growth [29]. To determine the impact of IK1 expression on the growth in MXP5 cells, we generated stable transduced MXP5 cells that express doxycycline (DOX) inducible IK1-IRES-GFP (IK1) or an empty vector IRES-GFP control (CON) (Figure 1A). MXP5 cells expressing IK1 grew slower over a 12-day period compared to control cells (Figure 1B), and IK1 induction arrested MXP5 cells in the G1 phase of the cell cycle 48 h after induction (Figure 1C). Thus, similar to PDX2 cells, IK1-induction results in a growth arrest in *IKZF1*-mutated MXP5 cells and triggers exit from the cell cycle. 

### 2.2. Identification of IK1-Regulated miRNAs

**Figure 2:**
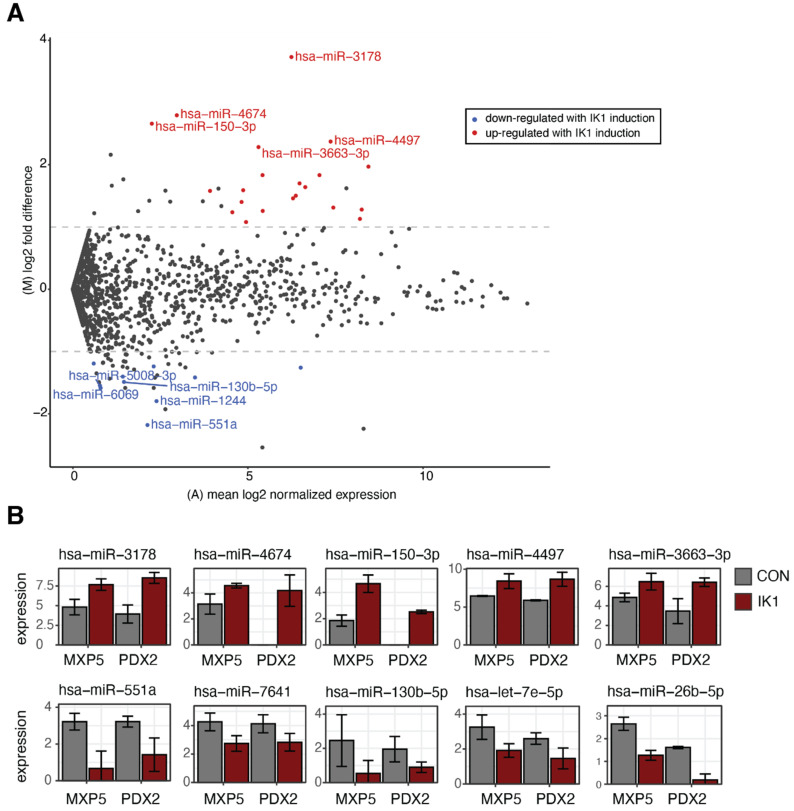
Global miRNA expression profiling of IK1-regulated miRNAs. (**A**) MA plot showing significantly differentially regulated miRNAs with IK1 induction in both MXP5 and PDX2 B-ALL cells. Significantly down-regulated (blue), up-regulated (red) miRNAs, and miRNAs not changed with IK1 induction compared to controls (black) are colored. The names for the top 5 miRNAs for both directions are shown. Statistical details are reported in Material and Methods. (**B**) Expression barplots showing the normalized expression values for 5 IK1-upregulated (top row) and 5 IK1-downregulated miRNAs (bottom row). Error bars, SEM; *n* = 2.

Because miRNAs are known to regulate cell growth in cancer, we investigated the changes in miRNA expression patterns associated with IK1-induced growth arrest in both MXP5 and PDX2 cells. We conducted miRNA expression profiling using microarrays that contain over 2500 human miRNAs, and found that 821 miRNAs were expressed above background in both B-ALL cell lines (Table S1). Differential expression analysis comparing 24 h of DOX induction in control and IK1-induced B-ALL cells identified a total of 31 differentially expressed miRNAs (DE miRNAs) (Figure 2A, Table S2). There were 21 upregulated and 10 downregulated DE miRNAs with IK1 expression compared to control. Among the identified IK1-regulated miRNAs, 5/31 unique miRNAs have been previously described as leukemia miRNAs based on their target genes disease assocations [30]. Interestingly, several IK1-upregulated DE miRNAs have been classified as ‘oncosuppressive’, including hsa-miR-150-3p [31,32], hsa-miR-3663 [33], and hsa-miR-4497 [34] (Figure 2B). We also found that many of the DE miRNAs that were downregulated with IK1 induction have known oncogenic roles or are known as “oncomiRs”, including hsa-let-7e [35,36,37], hsa-miR-551a [38], hsa-miR-7641 [39,40], and hsa-miR-130b [41,42]. These results indicate that IK1-induced growth suppression is coupled with changes in the expression of miRNAs with both oncogenic and tumor suppressive potential.

### 2.3. Ikaros Binds to miRNA Gene Regions in B-ALL

**Figure 3:**
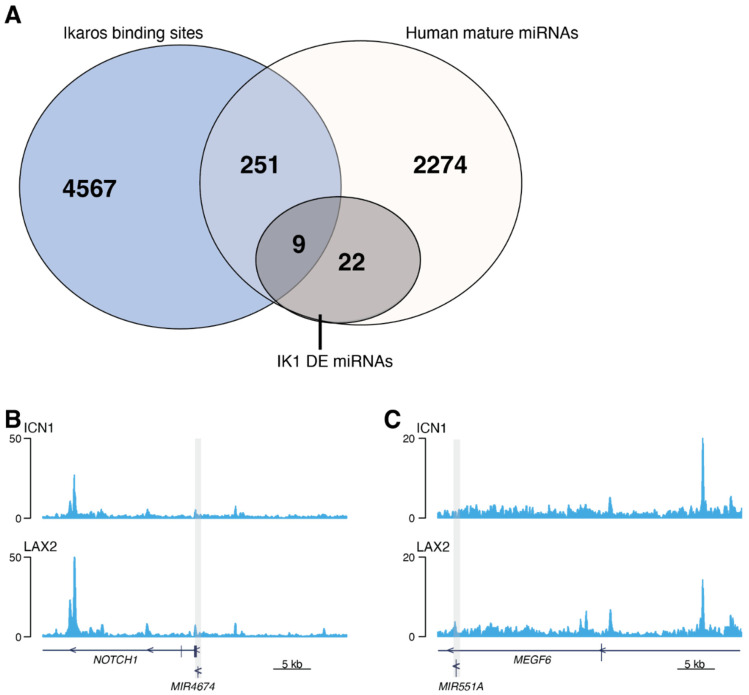
Ikaros binds to a subset of miRNA genes. Ikaros ChIP-seq from 2 Ph^+^ B-ALL cell lines with wild-type *IKZF1* (ICN1 and LAX2) were analyzed. (**A**) Venn diagram comparing the total number of Ikaros binding sites from B-ALL cell lines within 50 kb of all annotated human miRNA genes and the 31 significantly IK1 differentially expressed miRNAs. Genome browser snapshot of Ikaros ChIP-seq signal surrounding (**B**) the *MIR4674* locus encoding hsa-miR-4674 (chr9:136,526,173−136,566,259), and (**C**) the *MIR551A* locus encoding hsa-miR-551a (chr1:3,558,343−3,599,142). Locations of the miRNA genes are shaded. Shown are the read depth normalized fold enrichment (FE) ChIP-seq compared to input signal for both ICN1 and LAX2 datasets.

We next examined the occupancy of Ikaros at chromatin regions adjacent to the genes encoding primary miRNAs. We analyzed Ikaros ChIP-seq data from two independent human B-ALL cell lines with wild-type Ikaros expression [29], and annotated genes with transcription start sites (TSSs) within 50 kb of high-confidence Ikaros binding sites (4827 total sites). We found Ikaros binding within 50 kb of 260 unique genes encoding miRNAs (Figure 3A). Among the 31 miRNA that were differentially expressed with IK1 induction, nine were bound by Ikaros (29%) (Table S3). This included hsa-miR-4674 (encoded by *MIR4674*) upregulated by IK1, and hsa-miR-551a (encoded by *MIR551A*) down-regulated by IK1 (Figure 3B,C). Together, these results suggest that Ikaros has the potential to directly regulate the expression of a subset of miRNA genes in B-ALL cells.

### 2.4. miRNA-mRNA Regulatory Networks of IK1-Regulated miRNAs

**Figure 4:**
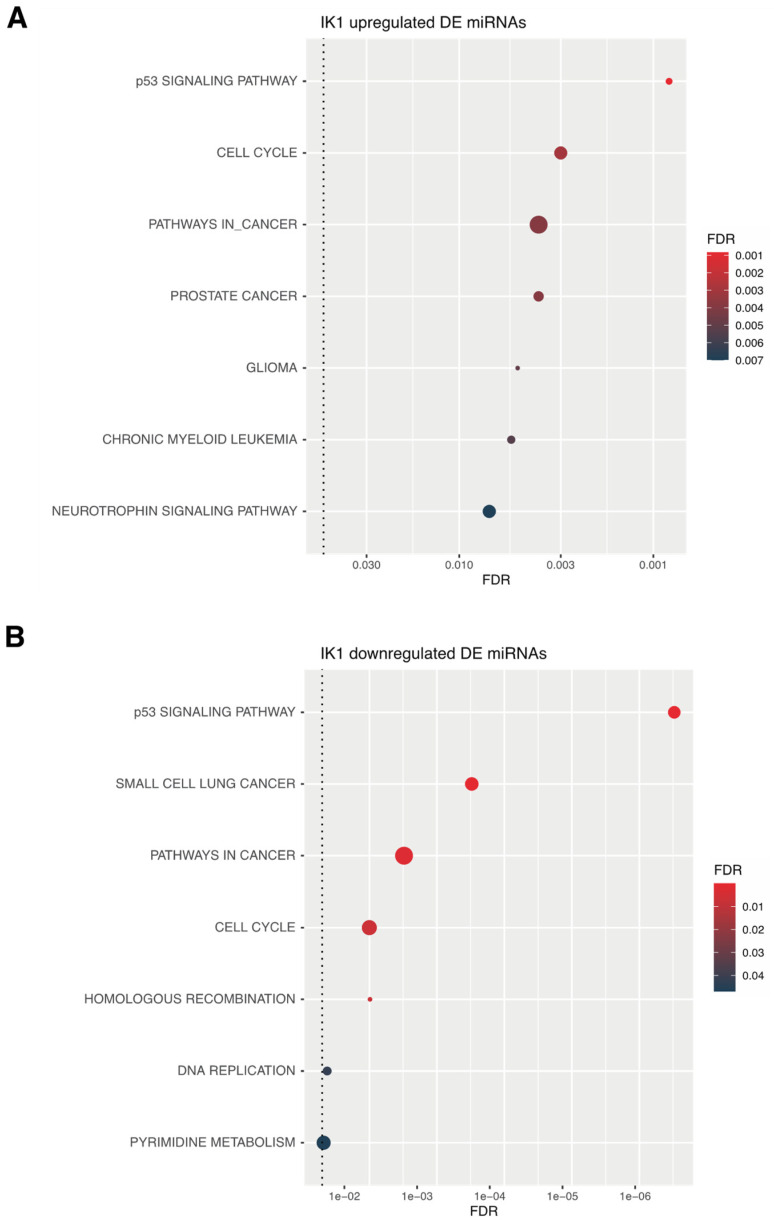
Pathway analysis of IK1-regulated miRNAs. KEGG pathway enrichment analysis for the validated targets of all (**A**) IK1 upregulated differentially expressed miRNAs or all (**B**) IK1 downregulated differentially expressed miRNAs. Shown are the top 13 enriched Gene Ontology Biological Process genesets, the dot color represents the significance (false discovery rate (FDR)) and the dot size signifies the geneset size.

Complex networks of miRNA and their target genes regulate diverse biological processes. An individual miRNA can have many different mRNA targets, and likewise, a single mRNA can be regulated by multiple different miRNAs. To determine the putative target genes of IK1-regulated miRNAs, we collected the experimentally validated mRNA targets of both up- and down-regulated IK1 DE miRNAs contained in the miRTarBase miRNA-target interactions database [43]. This revealed a total of 1542 and 3290 putative target genes for the 21 upregulated and 10 down-regulated miRNAs, respectively (Table S4). Pathway analysis revealed the significantly enriched KEGG pathways for these two groups of target genes (Table S5). The combined set of target genes for the group of 21 IK1-upregulated DE miRNAs were significantly enriched (false discovery rate (FDR) < 0.05) in biological processes including cell cycle, the p53 signaling pathway and pathways in cancer (Figure 4A). As the biological phenotype in our experimental setup was cell cycle arrest upon IK1 induction (Figure 1), we evaluated the target genes of the IK1 up-regulated miRNAs contained within the KEGG cell cycle geneset. In agreement with the observed growth arrest, we found several positive regulators of cell cycle among the targets of IK1-upregulated miRs (e.g., *CCDN1*, *CCDN2* and *CDK6*, targeted by hsa-miR-8069, hsa-miR-4516 and hsa-miR-3663-3p, respectively). Hence, Ikaros induced expression of miRNAs that regulate the expression of genes promoting cell cycle progression. 

**Figure 5:**
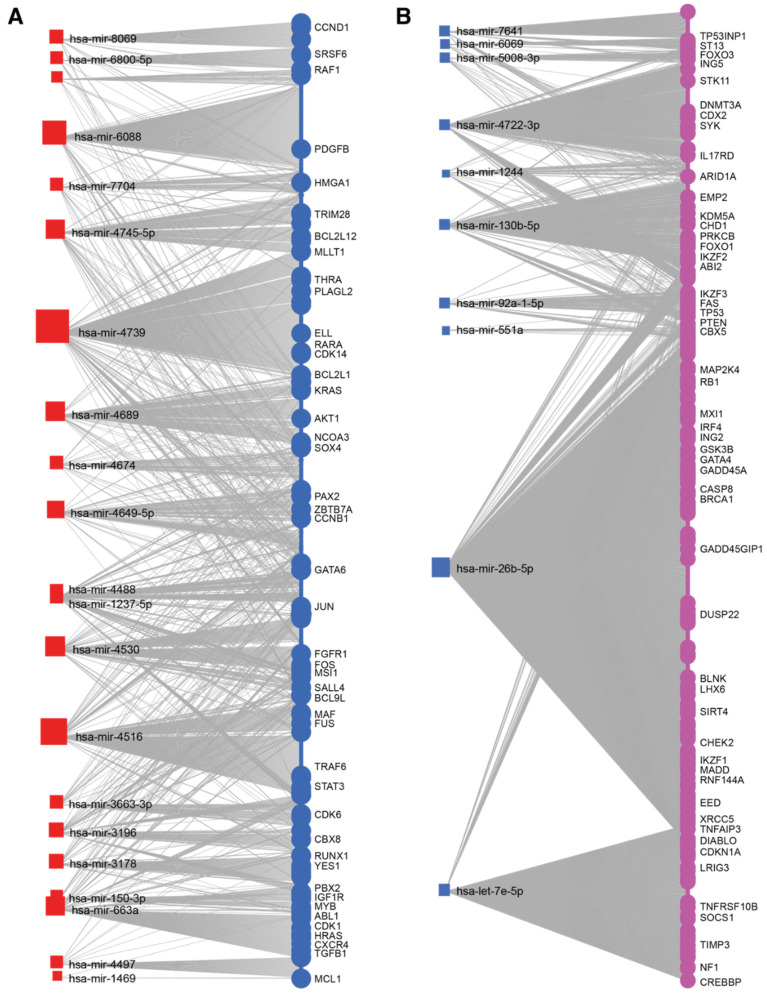
miRNA-mRNA target networks for IK1-regulated miRNAs. The collection of mRNA targets for (**A**) 21 upregulated DE miRNAs highlighting oncogenic targets, and (**B**) for 10 downregulated miRNAs, highlighting tumor suppressor gene targets. Size of the miRNA nodes (squares) correspond to the number of target genes in miRTarBase for different miRNAs. Only some of the oncogene (**A**) and tumor suppressor (**B**) target genes are listed. For full list, see Table S7.

Interestingly, the targets of the IK1-downregulated miRNAs were also enriched in the p53 signaling pathway, cell cycle, and pathways in cancer (Figure 4B). The common pathways between up- and down-regulated IK1 DE miRNA targets exemplified how several cell cycle and cancer-associated genes can be targeted by multiple different miRNAs. Therefore, we compared the target genes and filtered the data to obtain only the unique non-overlapping target genes of either up- and down-regulated DE miRNAs (1081 and 2829 genes, respectively) (Table S6). Among the non-overlapping target genes for IK1 up-regulated miRNAs, we found several known oncogenes, such as *ABL1*, *HRAS*, *KRAS*, and *MYB*. In fact, comparing with a curated list of oncogenes, we found that 89 of the target genes for IK1 up-regulated miRNAs were known oncogenes (Figure 5A and Table S7). Similarly, comparing with a list of identified tumor suppressor genes, we found that 219 of the target genes for IK1 down-regulated miRNAs were tumor suppressor genes (Figure 5B and Table S7). The non-overlapping targets of IK1 down-regulated miRNAs included the tumor suppressors *RB1*, *NF1*, *BLNK* and *GADD45A*, as well as *IKZF1* and the family member *IKZF2* (Figure 5B). Furthermore, the non-overlapping target genes of IK1 down-regulated miRNAs included genes associated with apoptosis and programmed cell death (i.e., *CASP3*, *CASP4*, *CASP7*, *CASP8*, *CASP9*) (Table S6), suggesting IK1 induction leads to relief of miRNA-repression of these genes. Taken together, we found that Ikaros regulates the expression of mutliple miRNAs that are know to target several critical regulators of oncogenic cell growth and tumor suppressor functions.

### 2.5. IK1-Regulated miRNA Expression Correlates with Overall Survival 

**Figure 6:**
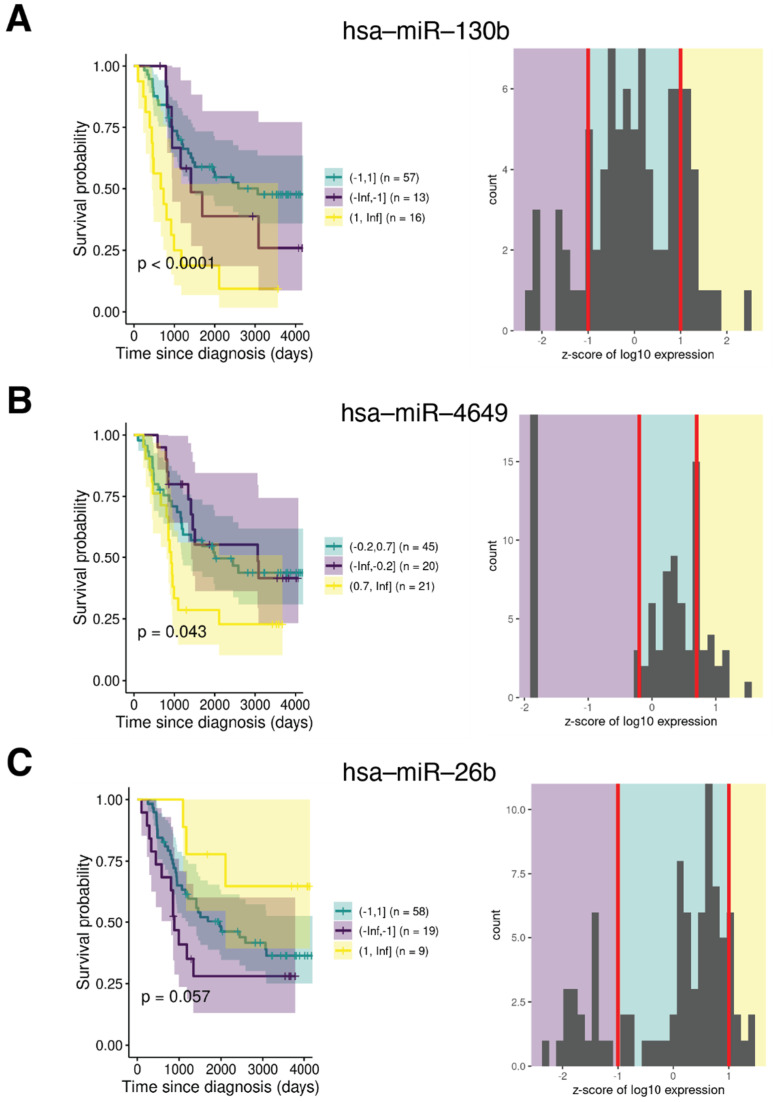
Association of miRNA expression with overall survival in a cohort of B-ALL patients. Kaplan–Meier survival analysis revealed the difference of overall survival rate between patients with different expression levels of the indicated IK1-regulated miRNAs, based on z-score binning. The survival probability of B-ALL patients with high expression of (**A**) hsa-miR-130b and (**B**) hsa-miR-4649 is worse than that of patients with low expression. The high expression of (**C**) hsa-miR-26b has positive effect on overall survival probability. Shown are the Kaplan- Meier survival curves with *p*-values indicated, as well as the z-score bins showing the normalized miRNA expression counts on the *Y*-axis.

We next evaluated the expression of the IK1-regulated miRNA genes as prognostic indicators in B-ALL. Using miRNA-seq data from a cohort of 86 primary bone marrow B-ALL specimens from the Therapeutically Applicable Research to Generate Effective Therapies (TARGET) project, we performed multivariate Cox analysis to evaluate the prognostic significance of the expression of IK1 DE miRNAs on overall survival (OS) [44]. Of the 31 total Ikaros-regulated DE miRNAs, the expression of 3 DE miRNAs showed significant correlation with OS in the TARGET dataset (Figure 6). The most significant miRNA (*p* < 0.0001) hsa-miR-130b was downregulated by IK1, and high expression of hsa-miR-130b correlated with overall worse outcome in this patient cohort of B-ALL (Figure 6A). In addition, the expression of another IK1-downregulated miRNA hsa-miR-26b and one IK1-upregulated miRNA hsa-miR-4649 were significantly associated with OS (*p*-value = 0.057 and *p* < 0.05, respectively). Patients with higher expression of hsa-miR-26b expression had a more favorable outcome, whereas patients with a higher expression of hsa-miR-4649 had a worse outcome in this B-ALL patient cohort (Figure 6B,C). 

## 3. Materials and Methods

### 3.1. Cell Culture 

Two *IKZF1*-mutated Ph^+^ B-ALL cell-lines (PDX2 and MXP5) were derived from previously described xenograft-expanded deidentified human B-ALL cells that were initially maintained in vitro on irradiated OP9 stroma cells [29,45,46,47]. These cells have adapted to in vitro growth without OP9 stroma cell support, and were maintained at 37 °C in humidified incubator with 5% CO_2_ in minimum essential medium alpha (MEMα) GlutaMAX (Gibco, Thermo Fisher Scientific, Waltham, MA, USA), supplemented with 20% FBS (Gibco, Thermo Fisher Scientific, Waltham, MA, USA).

### 3.2. Lentiviral Constructs and Transduction 

Cloning and cell line generation was performed as described previously [29]. Briefly, full length Ikaros isoform (Ik1) with IRES-GFP and an empty vector IRES-GFP control were cloned into the TET-inducible expression vector pLVX-TRE3G-IRES (Takara Bio, San Jose, CA, USA) and transduced into B-ALL cells expressing TET activator pLVX-EF1a-Tet3G (Takara Bio, San Jose, CA, USA). Expression of wild-type IK1 and GFP control samples was induced with the addition of 1 ug/mL doxycycline, a TET analog. 

### 3.3. Flow Cytometry and Sorting 

GFP signal was monitored using LSR Fortessa (BD Bioscience, Franklin Lakes, NJ, USA). For cell cycle analysis, cells were permeabilized with Cytofix/Cytoperm kit (BD Bioscience, Franklin Lakes, NJ, USA), stained with DAPI and gated on GFP^+^. GFP^+high^ cells were sorted using FACS Aria II (BD Bioscience, Franklin Lakes, NJ, USA). Data were analyzed using FlowJo and FACS Diva software. 

### 3.4. Immunoblot Analysis

Protein was extracted from induced cells and Western blot was performed as described previously [29]. Blots were probed with an antibody raised against N-terminal of Ikaros (residues 1–80) [48] and α-tubulin (Biolegend, San Diego, CA, USA, cat #627902).

### 3.5. RNA Isolation 

RNA was isolated using the Direct-zol RNA kit (Zymo Research, Irvine, CA, USA) following manufacturer’s instructions. RNA was then assessed for quality with the Agilent Bioanalyzer and quantified with the qubit fluorometer. 

### 3.6. Microarray Data and Differential miRNA Expression Analysis 

Purified RNA was used for hybridization on GeneChip™ miRNA 4.0 Arrays (ThermoFisher Scientific, Waltham, MA, USA) and processed as previously reported [49]. Briefly, raw CEL files were processed using the apt-probeset-summarize function within the Affymetrix Power Tools v1.18.0 (APT) software package (ThermoFisher Scientific, Waltham, MA, USA), and the Simplified Expression Analysis algorithm was used to summarize probesets and the Detectable Above BackGround (DABG) algorithm was used to assign *p*-values to probeset intensities. All annotation and library files for the miRNA v4.0 arrays were obtained from the manufacturer. Following probeset summarization, the average of 95 anti-genomic probesets was subtracted from each RNA expression probeset. Only probes for 2578 human mature miRNAs were selected for further analysis. Any value less than 0, below microarray background, was set to 0. Probesets with a DABG *p* > 0.05 were set to 0. These background normalized values were log2 transformed. To identify differentially expressed mature miRNAs, we compared MXP5 and PDX2 GFP control replicates to MXP5 and PDX2 IK1 replicates (each with independent duplicate samples) and applied Tukey’s HSD test with Benjamini and Hochberg correction for multiple testing. The differentially expressed miRNAs were identified according to the criteria of having a significant difference between mean signal for the CON and IK1 groups (Tukey’s HSD Difference > 1 and FDR corrected *p* < 0.05). This analysis was performed in the R 4.2.0 statistical environment.

### 3.7. Construction of miRNA-mRNA Networks and Pathway Enrichment Analysis

The mRNA targets of the differentially expressed miRNAs were collected from the miRTarBase v 8.0 [43] using the miRNetR package [50]. The miRNA-mRNA regulatory network was constructed based on the predicted and validated targets and visualized via Cytoscape software (version 3.4.0; www.cytoscape.org, accessed on 15 January 2021). Gene set enrichment analysis for the collection of all validated mRNA targets of either up- or down- differentially expressed miRNAs was performed against the KEGG geneset database [33] using the hypeR package with an false discovery rate (FDR) cutoff of less than 0.05 and a background size of 17,387 based on the number of target genes the miRTarBase v 8.0 [43]. We compared the list of IK1 up-regulated miRNA targets to a list of known oncogenes derived from ONGene [51], and similarly the list of IK1 down-regulated miRNA targets to a list of tumor suppressor genes derived from TSGene [52]. 

### 3.8. ChIP-Seq Analysis 

We reanalyzed previously generated Ikaros ChIP-seq data (NCBI Gene Expression Omnibus accession GSE58825) [29] by alignment to the hg38 genome using bowtie2 version 2.4.5 [53]. Significantly enriched peaks were called against input control using MACS2 [54]. Only peaks that were present in two independent replicates for two independent B-ALL cell lines (ICN1 and LAX2) were used for downstream processing of data. In total, 4827 Ikaros peaks were annotated within 50 kb of annotated hg38 gene transcription start sites using homer [55]. Fold-enrichment (FE) signal values of ChIP-seq versus input were obtained using MACS2 and visualized using the trackplot R package [56].

### 3.9. Survival Analysis

Processed miRNA sequencing data was downloaded from the Genomic Data Commons (GDC) data portal (https://portal.gdc.cancer.gov/, accessed on 1 March 2021), National Cancer Institute (NCI) TARGET: Therapeutically Applicable Research to Generate Effective Treatments, dbGaP Study Accession: phs000218.v24.p8. Samples with missing survival data were excluded from the analysis. Hazard ratio (HR), 95% confidence intervals (CI) and log-rank *p* values were calculated. We applied the “survival” R package v2.38 (http://CRAN.R-project.org/package=survival/, accessed on 1 March 2021) for Cox regression analysis and the “survplot” R package v0.0.7 (http://www.cbs.dtu.dk/~eklund/survplot/, accessed on 15 January 2021) for generating Kaplan–Meier plots. We determined each percentile of miRNA expression by using z-scores for expression as a cutoff point to divide patients into high, mid and low expression groups. 

## 4. Discussion

In this study, we used inducible expression of wild type IK1 in *IKZF1*-mutated human Ph^+^ B-ALL cell lines to study Ikaros tumor suppressor mechanisms in leukemia. Accordingly, IK1 induction in these cells resulted in growth arrest with cell cycle exit supporting the function of Ikaros as a tumor suppressor. Mechanisms attributed to Ikaros growth suppression in B-ALL include transcriptional regulation of a number of genes involved in various biological processes, including those that function in B cell development and act downstream of the pre-B cell receptor (pre-BCR) [29,57,58], as well as genes that limit the metabolic capacity of leukemia cells [45]. However, among the many transcriptional targets of Ikaros it remains unclear which are crucial for B-ALL growth suppression. Because miRNAs are implicated in B cell development [59], and have known oncogenic (oncomiRs) and tumor suppressor roles (tsmiRNAs) [60,61], we sought to investigate a potential role for Ikaros in the regulation of miRNA gene expression. 

*IKZF1* mutations are associated with poor prognosis and high recurrence rate in the Ph^+^ subtype of B-ALL [12,14]. We therefore established a system to identify IK1-regulated miRNAs based on the expression of IK1 in two independent *IKZF1*-mutated human Ph^+^ B-ALL cell lines (MXP5 and PDX2) and conducted global miRNA expression profiling by microarray analysis. Our results derived from biological replicates identified a total of 31 miRNAs that changed expression with IK1 growth suppression. Integrating differential miRNA expression with Ikaros ChIP-seq data revealed that the TSS regions (50 kb) of 9 Ikaros-responsive miRNAs were bound by Ikaros. Ikaros is known to regulate gene expression through recruitment of chromatin modifying complexes, including regulation of enhancer regions. Hence, the observed binding of Ikaros to these miRNA loci suggests that Ikaros can function to directly regulate the expression of these miRNAs. In the event of loss-of-function *IKZF1* mutations, as are observed in subtypes of B-ALL, these miRNA genes are subsequently deregulated. 

Ikaros is known to play critical roles in progenitor cells through the transcriptional regulation of stage-specific gene expression programs, and recognized Ikaros target genes include those encoding cytokines, cytokine receptors, and other factors central to progenitor cell proliferation and differentiation [62]. However, little is known about the role of Ikaros in the regulation of miRNA expression during lymphoid development. Here, we identified multiple Ikaros-regulated miRNAs in leukemia cells that have been previously suggested to function in B cell development, suggesting a potential role for Ikaros tumor suppression is to regulate developmental miRNA expression. More investigation is needed to understand the potential role for Ikaros to control the expression of developmental miRNA-mediated gene regulatory networks in normal B cell development and to understand how these become altered in leukemia. Comparably, an alternative Ikaros tumor suppressor role is to counteract distinctive oncogenic signals that lead to aberrant miRNA expression in leukemia. However, more studies are needed to understand the different oncogenic factors that contribute to aberrant miRNA expression in different types of leukemia, and to clarify the function of miRNAs in normal and malignant progenitor cells. 

miRNAs are known to be pleiotropic in the context of oncogenesis, where a miRNA may have both oncogenic and tumor suppressive roles [61]. We hypothesized that miRNAs that were upregulated with IK1 induction may represent putative tsmiRNAs, and studying their corresponding target networks may reveal potential tumor suppression processes connected to B-ALL. We identified a total of 21 miRNAs upregulated upon IK1 induction, several of which have known growth suppressive roles (Table S2). For example, we identified miR-150 as a miRNA upregulated by IK1 induction; several studies have reported miR-150 functions in the immune system, regulating the development of B-cells, T-cells and NK cells [63,64]. Notably, miR-150 has previously been demonstrated to be expressed at lower levels in B-ALL compared to healthy control, including in the poor-outcome subgroup of MLL-rearranged leukemia, and furthermore, miR-150 was found to be downregulated in relapsed B-ALL compared to B-ALL in clinical remission (CR) [65,66,67]. Other potential IK1-upregulated tsmiR include; miR-4497, which has been reported as a tumor suppressor in squamous cell carcinoma [34]; miR-3663-3p, which was reported as a tsmiR in hepatocellular carcinoma and to be targeting *CCND1* in gastric cancer [33,68]; and miR-3178, which was reported as a tsmiR in gastric cancer [69], was shown to be downregulated in hepatocellular carcinoma tumor endothelial cells and promoted the apoptosis and G1 phase arrest [70], and to represses DNA replication by repression of *CDC6* [71]. 

Similarly, based on decreased expression with IK1-induced growth arrest, we postulated that miRNAs downregulated with IK1 may be putative oncomiRs in B-ALL. IK1 induction downregulated the expression levels of 10 distinct mature miRNAs, many which have recognized roles in cancer. Notably, we identified miR-130b as being downregulated by IK1 induction. miR-130b is highly expressed in B-ALL [72], including the poor prognosis subgroup of MLL-AF4 Acute leukemia, and mechanistic studies have explored miR-130b as an oncomiR in other cancers, such as colorectal and lung cancer [41,65,73,74]. It has been suggested that miR-130b regulates growth arrest in B-lymphoid differentiation, and these mechanisms contribute to the direct downregulation of tumor suppressor genes *NR2F6* and *SGMS1* in MLL-rearranged leukemia [74]. We furthermore identified miR-92a as a miRNA downregulated by IK1 induction; miR-92a is derived from the polycistronic miR-17-92 cluster (also known as oncomiR-1) and has been shown to play oncogenic roles to silence the tumor suppressor genes *p53* and *GATA1* [75]. Additional IK1-downregulated miRNAs included miR-7641, which has been previously shown to be highly expressed in metastatic cancer cells and to promote tumor cell progression and metastasis [39]; let-7e that has been shown to have a role in ovarian cancer cisplatin resistance through the regulation of DNA damage repair target genes BRCA1 and Rad51 [76], as well as cisplatin-resistance factors *CCND1* and *EZH2* [35]; and miR-551a, for which increased expression has been shown to be protective against chemotherapy-induced cell death [38].

To study the potential biological roles of IK1-regulated miRNAs, we collected lists of experimentally validated target genes and performed pathway enrichment analysis and network visualization. KEGG pathway enrichment analysis revealed a similar enrichment of cell cycle and cancer pathways for the targets of both IK1-up and downregulated miRNAs (Figure 4). Closer inspection of the list of target genes revealed some overlap of target genes between the targets of upregulated and downregulated miRNAs, reflecting how genes can be targeted by multiple miRNAs. However, a comparison of the target genes for IK1-up and IK1-downregulated miRNAs showed hundreds of targets unique to either IK1 up- and IK1 down-regulated miRNAs (Table S6), including many which have recognized roles as oncogenes (e.g., *KRAS*, *HRAS*) and tumor suppressor genes (e.g., *GADD45A*, *TP53*, *BRCA1*, etc.) Among the unique target genes involved in cancer pathways is the *ABL1* gene that is a target gene of hsa-miR-663a, which was up-regulated with IK1 growth suppression. The Ph^+^ B-ALL cells used in the experimental model have constitutive expression of active ABL1 kinase through the BCR-ABL1 oncogene translocation. Hence, a possible tumor suppressive mechanism of Ikaros is to activate the expression of a miRNA (hsa-miR-663a) that represses ABL1 expression to restrict cell growth, and mutation in *IKZF1* in Ph^+^ B-ALL could relieve such oncogene repression by reduced tsmiR levels. While it remains to be determined if Ikaros regulates the expression of key growth targets via miRNA intermediates, these results suggest that Ikaros tumor suppression may include extensive miRNA-mRNA growth networks.

Aberrant miRNA expression has been previously shown to serve as prognostic indicators to predict treatment response or disease relapse for hematologic malignancies and cancers [77]. We analyzed small RNA-seq data generated in B-ALL patients by the TARGET project and found three IK1-regulated miRNAs were associated with outcome in this cohort. Notably, patient survival analysis showed that high expression of identified IK1-repressed miRNAs hsa-miR-130 was specifically associated with poor outcome and might represent a potential biomarker in B-ALL. Two additional miRNAs were identified, albeit with lower significance. These 3 miRNAs were among those DE miRNAs not bound by Ikaros in ChIP-seq data (Figure 3). The mechanism of how Ikaros regulates the expression of these is currently unknown. Furthermore, as the prognosis analysis was performed on a limited patient cohort with unknown *IKZF1*-status, it is possible that future analysis with larger patient cohorts, or analysis of specific leukemia subtypes, might uncover additional correlations between other IK1-regulated miRNAs and patient outcome. 

Herein, we have identified 31 miRNAs that are regulated by the tumor suppressor Ikaros in *IKZF1*-mutated Ph^+^ B-ALL cells. This included several miRNAs previously described as oncomiRs and tsmiRs, indicating that regulation of miRNAs is a part of Ikaros tumor suppressor function in B-ALL. Additional Ikaros-regulated miRNAs were identified that are less well-known in cancer, as well as miRNAs that have been described both as tsmiRs and oncomiRs in different cancer cell types. A limitation of this study was the use of microarray technology to identify Ikaros-regulated miRNAs. Because of its wider dynamic range as well as its capacity to identify a larger number of DE miRNAs, small RNA-sequencing protocols may generate more insight into mechanisms of Ikaros miRNA regulation [78]. Nevertheless, there is a gap in our knowledge of miRNA expression and biological function in different B-ALL subtypes. As more comprehensive miRNA expression datasets become available, from both patient samples and cell lines, it will be important to address the question of subtype-specific patterns of miRNA expression and their roles in different types of B-ALL. The roles of miRNA in hematopoiesis and leukemogenesis is an emerging field, and the biological functions of many miRNAs in leukemia remain unknown. Future work will focus on refining our understanding of the mechanisms modulated by the identified miRNAs and to determine whether they are key transcriptional targets of Ikaros tumor suppression in Ph^+^ B-ALL.

## Data Availability

Raw and processed miRNA expression data can be found in NCBI GEO accession GSE209694.

## Supplementary Materials

The following supporting information can be downloaded at: https://www.mdpi.com/article/10.3390/epigenomes6040037/s1, Table S1: miRNA expression values in B-ALL cells. Table S2: Differential miRNA expression analysis with IK1 induction compared to CON. Table S3: Ikaros-bound DE miRNAs. Table S4: Target genes of IK1 DE miRNAs. Table S5: KEGG pathway enrichment of all experimentally validated targets of IK1 DE miRNAs. Table S6: Non-overlapping target genes of IK1 upregulated and downregulated DE miRNAs. Table S7: IK1 downregulated miRNA targets that annotate as tumor suppressor genes and targets of Ik1 upregulated miRNAs that annotate as oncogenes.
