# Supplementary material for: Ikaros Regulates microRNA Networks in Acute Lymphoblastic Leukemia
Source: Epigenomes. 2022 Oct 19;6(4):37. doi: 10.3390/epigenomes6040037 (PMC9624360; doi:10.3390/epigenomes6040037)
Supplement: Supplementary file 1 [file epigenomes-06-00037-s001.zip › Table S3.pdf]

**IK-bound miRs:**	**Log2FC direction**
hsa-miR-1237	up
hsa-miR-4488	up
hsa-miR-4674	up
hsa-miR-4739	up
hsa-miR-6789	up
hsa-miR-6800	up
hsa-miR-551a	down
hsa-miR-1244	down
hsa-miR-4722	down
